# Online-Delivered Over Staff-Delivered Parenting Intervention for Young Children With Disruptive Behavior Problems: Cost-Minimization Analysis

**DOI:** 10.2196/30795

**Published:** 2022-03-11

**Authors:** Justin B Ingels, Phaedra S Corso, Ronald J Prinz, Carol W Metzler, Matthew R Sanders

**Affiliations:** 1 Department of Health Policy and Management College of Public Health University of Georgia Athens, GA United States; 2 Office of Research Kennesaw State University Kennesaw, GA United States; 3 Center for Research on Child Well-Being University of South Carolina Columbia, SC United States; 4 Oregon Research Institute Eugene, OR United States; 5 Parenting & Family Support Centre Brisbane Australia

**Keywords:** online parenting intervention, child disruptive behavior problems, cost-minimization analysis, online versus staff delivery, evidence-based parenting support, population reach

## Abstract

**Background:**

High-prevalence childhood mental health problems like early-onset disruptive behavior problems (DBPs) pose a significant public health challenge and necessitate interventions with adequate population reach. The treatment approach of choice for childhood DBPs, namely evidence-based parenting intervention, has not been sufficiently disseminated when relying solely on staff-delivered services. Online-delivered parenting intervention is a promising strategy, but the cost minimization of this delivery model for reducing child DBPs is unknown compared with the more traditional staff-delivered modality.

**Objective:**

This study aimed to examine the cost-minimization of an online parenting intervention for childhood disruptive behavior problems compared with the staff-delivered version of the same content. This objective, pursued in the context of a randomized trial, made use of cost data collected from parents and service providers.

**Methods:**

A cost-minimization analysis (CMA) was conducted comparing the online and staff-delivered parenting interventions. Families (N=334) with children 3-7 years old, who exhibited clinically elevated disruptive behavior problems, were randomly assigned to the two parenting interventions. Participants, delivery staff, and administrators provided data for the CMA concerning family participation time and expenses, program delivery time (direct and nondirect), and nonpersonnel resources (eg, space, materials, and access fee). The CMA was conducted using both intent-to-treat and per-protocol analytic approaches.

**Results:**

For the intent-to-treat analyses, the online parenting intervention reflected significantly lower program costs (*t*_168_=23.2; *P<*.001), family costs (*t*_185_=9.2; *P<*.001), and total costs (*t*_171_=19.1; *P<*.001) compared to the staff-delivered intervention. The mean incremental cost difference between the interventions was $1164 total costs per case. The same pattern of significant differences was confirmed in the per-protocol analysis based on the families who completed their respective intervention, with a mean incremental cost difference of $1483 per case. All costs were valued or adjusted in 2017 US dollars.

**Conclusions:**

The online-delivered parenting intervention in this randomized study produced substantial cost minimization compared with the staff-delivered intervention providing the same content. Cost minimization was driven primarily by personnel time and, to a lesser extent, by facilities costs and family travel time. The CMA was accomplished with three critical conditions in place: (1) the two intervention delivery modalities (ie, online and staff) held intervention content constant; (2) families were randomized to the two parenting interventions; and (3) the online-delivered intervention was previously confirmed to be non-inferior to the staff-delivered intervention in significantly reducing the primary outcome, child disruptive behavior problems. Given those conditions, cost minimization for the online parenting intervention was unequivocal.

**Trial Registration:**

ClinicalTrials.gov NCT02121431; https://clinicaltrials.gov/ct2/show/NCT02121431

## Introduction

The most prevalent mental health problems in childhood require effective interventions that are deliverable with sufficient population reach in a cost-efficient manner. This need is especially true of early-onset disruptive behavior problems (DBPs), which pose a significant public health challenge. Approximately 10-15% of preschoolers and children at school entry exhibit at least mild to moderately severe DBPs [1]. Early-onset DBPs elevate the risk of a range of adverse outcomes such as subsequent mental health problems, academic failure, substance misuse, delinquency, risky sexual behavior in adolescence, and chronic mental health problems and life consequences in adulthood [2-5]. Parenting and family-focused interventions provide the most robust evidence-based prevention and treatment for DBPs across several contexts and child/family populations [6-9]. Due to the high prevalence of DBPs, there is a substantial need for services; however, too few children with DBPs receive such interventions despite intervention efficacy. Contributing factors include strained resources, understaffing, and low program availability on the programmatic side, while parents encounter barriers to participation, including transportation, childcare, work schedules, and perceived stigma [10,11]. Therefore, the expansion of intervention strategies beyond traditional delivery methods is essential to meet these needs.

Internet delivery of evidence-based parenting interventions for child DBPs could potentially improve the reach of these interventions [12,13]. Therefore, a noninferiority trial was conducted to test whether an online-delivered parenting intervention, derived from the evidence-based Triple P-Positive Parenting Program, performed as well as a staff-delivered version of the same program in addressing child DBPs. The trial involved randomization of 334 children (aged 3-7 years) with clinical levels of DBPs, and their families, to the two intervention arms. DBPs assessed by both independent observation and parental reports defined the primary outcome. Details and results of the trial are reported elsewhere [14]. The main finding was that the online intervention substantially reduced child DBPs to a comparable extent as the staff-delivered intervention. During the trial, pertinent cost data were collected on both interventions and provided the basis for this study.

A cost-minimization analysis (CMA) was determined to be the most appropriate form of economic evaluation for assessing an intervention option that is noninferior in its primary outcome [15]. From this perspective, if two interventions produce similar effects, the less costly option is favorable. This method is standard in pharmaco-economics when comparing two clinically effective and equivalent therapies. While CMA is less common in other disciplines, it has been recognized as an appropriate method for comparing interventions delivered through technology-based methods against in-person delivery formats [16]. Previous work has identified the importance of evaluating internet-based interventions' costs, not just to the provider but also to those costs that fall on the user [17,18].

## Methods

This study's objective is to examine the costs of an online parenting intervention for childhood DBPs compared with a staff-delivered version of the same intervention. A CMA was completed in both intent-to-treat (ITT) and per-protocol (PP) contexts to achieve this objective.

### Description of the Online and Comparison Interventions

The online-delivered intervention (ODI) was Triple P Online, derived from the Triple P-Positive Parenting Program system of parenting interventions [19,20]. The ODI content draws on 17 core Triple P positive parenting skills and seeks to promote parental self-regulation. Examples of covered topics include understanding the causes of children's behaviors, strategies for fostering child development and skill acquisition, managing misbehavior effectively, planning to prevent problems, preparing for potential relapses of problematic behavior, and maintaining changes over time. The ODI incorporates video modeling of principles and specific parenting strategies, concrete tasks for parents to undertake with their children, and opportunities to engage in goal setting, constructive self-evaluation, and improvement. Structurally, the ODI consists of 8 modules, which are sequenced and take approximately 45-60 minutes each. The program includes easy navigation, video excerpts, personalized elements (eg, goal setting, content review, feedback, and a customizable workbook), interactive exercises, and downloadable worksheets. Following baseline assessment and randomization, the parent was shown how to access the online program and received a succinct orientation. During ODI implementation, a staff member made brief contact with the parent by phone, email, or text at 2, 4, 8, and 13 weeks to check on technical problems and prompt utilization of the program but did not provide any content coaching or clinical assistance.

The comparison was the staff-delivered intervention (SDI), Level 4 Standard Triple P-Positive Parenting Program, which involves 10 in-person 60-75-minute sessions delivered by a trained and accredited practitioner. The SDI parallels the ODI in terms of parenting principles and strategies imparted, promotion of parental self-regulation, video modeling within a session, and between-session practice activities for parents.

### Study Sample and Clinical Trial Design

The sample consisted of 334 families of children ages 3-7 years who exhibited pronounced levels of oppositional and disruptive behaviors: 63% (212/334) boys and 37% (122/334) girls. The racial distribution for the parents was: 63% (210/334) non-Hispanic White, 21% (69/334) African American, 8% (27/334) Hispanic White, and 8% (27/334) other races. The families included 69% two-parent (230/334) and 31% (104/334) one-parent households. For educational attainment of the participating parents, 14% (47/334) had high-school graduation or less, 28% (92/334) some college, and 57% (192/334) college graduation.

The families were enrolled in a clinical trial in which they were randomly assigned to either the ODI or the SDI for the goal of acquiring positive parenting strategies to reduce child DBPs and improve child and family functioning. Characteristics of the trial included demographic and baseline equivalence across conditions, multi-source outcome measures (ie, observers, parents, and teachers), post-intervention and follow-up (12 months after baseline) outcome assessments, independent assessment of intervention fidelity, and analyses using both ITT and PP methods. A full description of the trial and outcomes is reported elsewhere [14]. The study design for the costing analysis was built on this randomized clinical trial.

The full sample consisted of 168 (50%) and 166 (50%) families for the ODI and SDI groups, respectively; all included in the ITT analysis. The PP-based costing analysis included the 54 (34%) ODI and 106 (66%) SDI families. They had completed their assigned program in its entirety, which met a conservative completion threshold even though other families excluded from the PP sample had completed most but not all of the programs. The critical consideration for this cutoff is to ensure that the PP analysis captures the costs of each intervention when taken to completion.

### Study Design for the Costing Analysis

The costing analysis goal was to determine if there was a significant difference in the resources required to administer and deliver the ODI compared with the SDI. The study team designed the collection and analysis of resource utilization data to estimate the incremental differences between the online and staff-delivered versions of the intervention. The identification and measurement of resource utilization were guided by program and participant perspectives.

As is typically the case for parenting interventions with children and families, the bulk of resource utilization was personnel-related, especially for the SDI condition. As a result, the burden of data collection fell on intervention staff who regularly completed written logs to document all time spent on intervention delivery and administration, including clinical supervision. Personnel recorded their time on these logs in 15-minute increments and placed each time segment into one of several activity categories provided on each time log. For family-specific time, staff indicated time for a specific family/case. Intervention staff recorded administrative and supervisory time without reference to any particular family/case. Additionally, each SDI parent completed a brief form to document resource utilization related to their family's participation. These forms were either mailed directly or scanned and emailed to the cost team, where graduate assistants coded the logs into Excel spreadsheets.

Table 1 delineates the activity categories included in each log for each intervention. The direct resource category comprises intervention delivery and communication with each family, including activities before, during, and after intervention sessions. The administrative resource category refers to activities attributable to the intervention (eg, professional supervision) but not to any specific family. The nondirect resource category consists of personnel time spent on behalf of families outside of intervention-session delivery, including travel, waiting on families, and communication about families.

**Table 1:** Staff activities and resource categories captured on family-specific and administrative log forms in the implementation of the online-delivered (ODI) and staff-delivered (SDI) interventions.

Activity category	Resource category	Family-specific log			Administrative log	
		SDI	ODI	SDI		ODI
Preparation for session	Direct	√				
In-person session	Direct	√				
Orientation session	Direct		√			
Documentation task	Direct	√	√			
Other contact	Direct	√	√			
Peer supervision	Administrative			√		
Individual supervision	Administrative			√		
Staff meeting	Administrative					√
Other meeting	Administrative			√		√
Consultation with techs	Administrative					√

Parents receiving the SDI completed a brief meeting form for every session. Parents documented the mode of transportation to and from the session, miles traveled in a personal vehicle, work hours lost to participate, and any other expenses incurred by the family to participate (eg, childcare for other children in the family).

Nonpersonnel resource consumption for the cost analysis included personnel travel costs, meeting space, and office space. Space used for session delivery and other related intervention tasks was also used for purposes other than the ODI and SDI. Therefore, information was collected about the space, and personnel time was used as the driver for the value of space. Finally, two other nonpersonnel costs were added, including $15 per SDI family for a workbook (Materials category) and $50 per ODI family for a fee paid to use the online system (Fees category).

The following procedures were followed to estimate total costs from personnel and nonpersonnel resources. All SDI personnel time was valued at $44 per hour, while ODI direct and nondirect time was valued at $44 and administrative time at $32 per hour. All adult family time from intervention participation was valued at $20 per hour. Participant travel was valued at $0.55 per mile. Space utilized for SDI delivery, SDI and ODI program introduction (orientation), and support of delivery and administrative personnel was valued at $0.096 per square foot hour of personnel time based on typical local rates for office space rental. The space used for the SDI sessions and SDI/ODI orientation was 102 square feet, for other SDI personnel was 126 square feet, and for other ODI personnel, time was 112 square feet. Resource consumption data were collected from 2014 to 2017, and salaries reflect an average of how personnel were compensated during this period. All costs were valued or adjusted in 2017 US dollars. Families were in the program for less than one year; therefore, no discounting of costs is included in these estimates. For the primary cost analyses, all families were included regardless of whether a family completed the intervention, which preserved the ITT design. The second set of cost analyses was conducted to gauge the PP costs of the two interventions, including only the families who completed their assigned intervention.

### Ethics Approval

Ethical approval for this study was obtained from the University of South Carolina Institutional Review Board on April 11, 2013 (reference Pro00024933).

## Results

### Personnel Time

To summarize direct, nondirect, and administrative personnel time spent on the intervention, the time reported on all logs was summed by intervention and divided by the number of families participating in each intervention. Table 2 provides a breakdown of the personnel time per family, as reported by intervention staff. On average, personnel spent 17 hours more delivering and administering the SDI than the ODI, most of the difference occurring in the direct personnel time resource category (13.5 more hours). This additional time occurred not just from session delivery (8.2 more hours) but also from other direct times, including preparation, communication with families, and documentation (5.3 more hours). Most of the remaining difference in total personnel time was from 2.6 additional hours per SDI family spent on administrative tasks with less than an hour spent on nondirect tasks.

**Table 2:** Personnel time per family case (in hours) for online-delivered (ODI) and staff-delivered (SDI) interventions.

Resource category		Intervention Format			Incremental difference (SDI-ODI)		
			SDI (n=166)	ODI (n=168)			
**Direct personnel time**			15.9	2.5		13.4	
	Session delivery time		8.3	0.0		8.3	
	Other direct time		7.6	2.5		5.1	
Nondirect personnel time			0.6	<0.1		0.6	
Administrative personnel time			3.0	0.6		2.4	
Total personnel time			19.5	3.1		16.4	

### Family Time

Total SDI family time spent engaging in the intervention was estimated from personnel-reported logs for sessions. For the ODI, family time spent engaging in the intervention was based on a backend database linked to the delivery platform, which tracked the parent's time logged into the Triple P Online program. On average, SDI families spent 10.5 hours of their own time receiving intervention sessions in-person, while ODI families spent 7.7 hours of their own time receiving the intervention online. Participation in SDI also required participants to travel to the session location. On average, SDI families reported 104 total miles of travel to and from intervention sessions using personal vehicles. Families did not report travel by taxi or bus. Few SDI families (4%) indicated the need to miss work to attend intervention sessions for an average of 10 hours lost per family that reported any time missed and 0.4 hours per family overall. SDI families did not indicate a need to pay any other expenses not included in travel.

### Total Costs

The total costs per family for SDI and ODI, including personnel and nonpersonnel resources and participant resources, are delineated in Table 3 for the full sample preserving the ITT design. In general, the SDI had significantly higher costs per family when compared with the ODI. Incremental cost differences per family for the SDI over the ODI were $903 for program costs (*t*_168_=23.2; *P<*.001); $262 for family costs (*t*_185_=9.2; *P<*.001); and $1164 for total costs (*t*_171_=19.1; *P<*.001). Incremental program costs, found at the bottom of Table 3, include program costs (personnel and nonpersonnel resources), family costs, and total costs combining both. All costs were valued or adjusted in 2017 US dollars.

**Table 3:** Comparison of staff-delivered (SDI) and online-delivered (ODI) interventions for program, family, and total costs per case on an intent-to-treat basis.^a^

Resource category (costs per family case)					Intervention format							*t* test (df)				*P* value
					SDI (n=166)			ODI (n=168)								
**Per-family program costs**																
	**Personnel**															
		Direct	$699			$107										
		Nondirect	$26			$0										
		Administrative	$131			$19										
		Total personnel costs	$856			$127										
	**Nonpersonnel**															
		Space	$215			$12										
		Fees	$0			$50										
		Materials	$15			$0										
		Travel	$10			$0										
		Total nonpersonnel costs	$240			$62										
Total program costs					$1091 (SD $499)			$188 (SD $51)			23.2 (168)				<.001	
**Family costs**																
	Time			$297			$144									
	Other: travel			$104			$0									
	Other: lost work			$8			$0									
Total family costs					$405 (SD 357)			$144 (SD 89)			9.2 (185)				<.001	
Total costs per family (program + family costs)					$1496 (SD 778)			$332 (SD 108)			19.1 (171)				<.001	

^a^All costs were valued or adjusted in 2017 US dollars.

The same cost analysis was repeated focusing exclusively on completer cases in a PP basis and is delineated in Table 4. Incremental cost differences for the SDI over the ODI were $1171 for program costs (*t*_114_=34.0; *P*<.001); $312 for family costs (*t*_116_=9.2; *P*<.001; and $1483 for total costs (*t*_115_=26.4; *P*<.001).

**Table 4:** Comparison of staff-delivered (SDI) and online-delivered (ODI) interventions for program, family, and total costs per case on a per-protocol basis (completer cases only).^a^

Resource category (costs per family case)				Intervention format				*t* test (df)		*P* value
				SDI (n=106)		ODI (n=54)				
**Per-family program costs**										
	**Personnel**									
		Direct	$903		$102					
		Nondirect	$31		$0					
		Administrative	$131		$19					
		Total personnel costs	$1062		120					
	**Nonpersonnel**									
		Space	$266		$12					
		Fees	$0		$50					
		Materials	$15		$0					
		Travel	$10		$0					
		Total nonpersonnel costs	$291		$62					
Total program costs				$1353 (SD 347)		$183 (SD 52)		34.0 (114)		<.001
**Family costs**										
	Time			$412		$247				
	Other: travel			$142		$0				
	Other: lost work			$8		$0				
Total family costs				$559 (SD 340)		$247 (SD 55)		9.2 (116)		<.001
Total costs per family (program + family costs)				$1912 (SD 564)		$430 (SD 91)		26.4 (115)		<.001

^a^All costs were valued or adjusted in 2017 US dollars.

## Discussion

### Principal Results

This study provides some of the first data directly comparing resource investments for internet-delivered versus standard staff-delivered behavioral interventions in which programmatic content is held constant. This comparison is important considering the previously reported confirmation that the internet-delivered intervention (Triple P Online) is as efficacious as the well-established, evidence-based standard intervention (Level 4 Standard Triple P) in achieving significant reductions in child behavior problems. The main economic finding is that the internet-delivered program costs were significantly less than the standard staff-delivered program. This cost differential stems from a much smaller investment required for the internet-program provision and a lower burden on internet-program participants. Personnel costs were the most significant drivers of the difference between the delivery modalities. The personnel activities related to direct personnel time, including session delivery and delivery-support tasks such as preparation and documentation, were the most significant drivers of the difference. Internet-program participants reflected a lower burden because of less programming time and travel-related costs.

When comparing interventions from an economic perspective, cost-effectiveness is much more common than CMA because incremental effectiveness and costs are taken into account. In this study, however, CMA is more suitable because the two interventions were comparably efficacious [14], which obviates the need for cost-effectiveness analysis. The apparent simplicity of CMA should not detract from the fact that it rests on the same theoretical underpinnings as more complex economic evaluation methods such as cost-effectiveness analysis [21].

Internet-delivered interventions undoubtedly have the potential to reach a large number of persons in the population, conceivably leading to large-scale positive changes in preventing and reducing childhood problems for a relatively small investment [22] through the provision of evidence-based parenting support [23-25].

Internet-delivered interventions provide an alternative method for families to receive needed evidence-based services with the potential to overcome obstacles to in-person delivery. The flexibility of access promotes a learner-centered approach, enabling participation at a time that suits the parent. Although there is still a cost for online delivery associated with participant time, it is possible that given the flexibility of when this time is expended, that time comes at a lower cost to the participant than the more constrained scheduling of time in staff delivery. Internet-delivered interventions take on even greater importance when in-person delivery is too difficult or even unsafe such as during a pandemic or adverse weather events that might preclude safe travel to clinical services but do not disrupt internet access.

### Comparison With Prior Work

Two problems sometimes encountered with internet-based interventions are low participation rates and high dropout [26-28]. These problems could bias the costs of an internet-based intervention toward being less costly. However, this issue did not bear out with this study, in which cost minimization was greater for PP over ITT analysis. Had high dropout from the ODI biased the results, cost minimization would have been greater for ITT, which retained dropouts in the analysis.

### Strengths and Limitations

In a CMA, the most careful consideration of costs is typically confined to categories expected to differ between modalities rather than a complete accounting of all implementation costs. In this study's CMA, all the main program costs for both delivery modalities were likely captured in the analysis. The data describe not only cost minimization but also an accurate estimate of each program's cost. This study was limited to just over 150 participants in each modality. If the program were scaled to include a larger number of participants and implemented similarly as in this study, the cost per family in the ODI would likely stay roughly the same without escalation of administrative costs from scaling up. It is less clear how costs for the SDI might escalate when scaling up.

It is often contended that while the costs of implementing an internet-delivered program are expected to be lower than the staff-delivered counterpart, the development or upfront costs are often higher for such a program. For example, the internet-delivered program in this study required only a modest access fee for implementation. However, program delivery utilized an already developed platform, which did not enter the cost analysis. Development costs were not included in the CMA. However, it is not necessarily the case that the internet program's development costs exceeded those for the staff-delivered program. This study's standard staff-delivered program (Level 4 Standard Triple P) went through more than two decades of content, materials, and component development and validation studies, which undoubtedly contributed substantial costs to program development. The initial training of program personnel similarly contributed to upfront costs that did not enter the CMA.

In some contexts, the inclusion of the development costs might initially suggest that an online-delivered intervention is more costly until a large enough number of individuals receive the intervention to make up for those higher upfront costs. This should not be considered a limitation for this study as the cost of accessing the online program was included in the CMA. A related issue pertains to ongoing developmental costs. Costs can be incurred to update or modify online programs to refresh video content, accommodate platform changes, and keep up with technological advances such as artificial intelligence. Although perhaps not as obvious, in-person programs can also incur costs to remain contemporary and evidence-based.

Two additional limitations relate to potentially peripheral or optional costs. The first involved the availability of onsite childcare during SDI sessions. It is debatable whether this childcare cost during intervention delivery should be attributed to the program costs since the protocol does not specifically reference childcare, and many families did not use it. Given that onsite childcare costs were neither tracked nor included in the CMA, the reported cost differential is likely a conservative estimate that would have been larger if those costs had been included. The second optional cost pertains to the brief telephone contacts by staff to check on technical problems and prompt utilization for the ODI, which were not prescribed in the online program but were included in the present cost evaluation. Had this cost been left out, the ODI direct personnel costs (mean of $107/family) would have been lower, further increasing the SDI-ODI cost differential.

The proportion of completers in ODI was about half of that in SDI. Several factors might have contributed to this lower completion rate. These include the possibility that parents who have achieved their intended goals, in the absence of a practitioner setting appointment times and creating an expectation that session attendance is necessary, might find it easier to discontinue. There are no sanctions for an early exit from the online-delivered program. Session completion could potentially be improved by providing at least some professional phone support during the intervention [29]. However, the provision of professional support increases delivery costs without necessarily improving child outcomes. However, professional support can potentially improve session completion and child outcomes when a parent enters the program with mental health concerns and low self-efficacy (eg, depression) [27]. Some parents prefer to do the online program independently and are not seeking additional professional support, nor do they accept it when offered.

Within the Triple P system, although Level 4 Standard Triple P is the individual program recommended for children with significant conduct problems, it is possible that briefer lower-intensity versions might also benefit some children. These lighter-touch, low-intensity programs such as Level 2 Positive Parenting Seminars, Level 3 Primary Care Triple P, and Level 3 Brief Discussion Groups have been shown to work primarily as preventive interventions rather than as interventions for children with more severe conduct problems. These briefer variants with fewer sessions are disadvantaged by reduced opportunities for parent coaching and at-home practice.

### Conclusions

The online-delivered parenting intervention in this randomized controlled trial produced substantial cost minimization compared with the staff-delivered intervention that provided the same content. The mean differential for total costs was $1164 per case for the intent-to-treat analysis containing all cases and $1483 per case for the per-protocol analysis containing only cases where the family completed its assigned intervention. Cost minimization was driven primarily by personnel time and, to a lesser extent, by facilities costs and family travel time. The CMA was accomplished with three critical conditions in place: (1) the two intervention delivery modalities (ie, online and staff) held parenting intervention content constant; (2) families were randomized to the two parenting interventions; and (3) the online-delivered intervention was confirmed to be non-inferior to the well-established evidence-based staff-delivered intervention in significantly reducing the primary outcome, child disruptive behavior problems. Given those conditions, cost minimization for the online parenting intervention was unequivocal.

## Abbreviations

CMA: cost-minimization analysis
DBP: disruptive behavior problem
ITT: intent-to-treat
NIH: National Institute of Health
NIMH: National Institute of Mental Health
ODI: online-delivered intervention
PP: per-protocol
SDI: staff-delivered intervention
TPI: Triple P International
UQ: University of Queensland
